# Supplementary material for: Positive density dependence acting on mortality can help maintain species-rich communities
Source: eLife. 2020 Jun 18;9:e57788. doi: 10.7554/eLife.57788 (PMC7302881; doi:10.7554/eLife.57788)
Supplement: Supplementary file 1. [file elife-57788-supp1.pdf]

Supplementary file for:

Positive density dependence acting on mortality can help maintain species-rich communities

Thomas G. Aubier

Contents

Page 2: Supplementary file 1A – Differential equations: comparison with the usual Lotka-Volterra model and scaling of differential equations

Pages 3 - 11: Supplementary file 1B – Analytical derivation of the two-species model with asymmetric competition for resources

Page 12: Supplementary file 1C – Numerical method for analyzing the coexistence equilibrium in two-species models

Page 13: Supplementary file 1D – Simulations of the multi-species models

Supplementary file 1A: Differential equations: comparison with the usual Lotka-Volterra model and scaling of differential equations

The final dynamical systems are obtained after rescaling all variables to growth rate and carrying capacity. Here, I describe the original two-species model with asymmetric competition for resources and I present the detail of the scaling process leading to Equation 1a in the main text.

1 Differential equations

The systems of differential equations describing the changes in density of the two species (N_1 and N_2) are:

$$\begin{cases} \dfrac{\mathrm{d}\,N_1}{\mathrm{d}\,\tau} &= r\,N_1\left(1-\dfrac{N_1}{K}\right)-\dfrac{m}{1+S\,N_1}\,N_1 \\ \dfrac{\mathrm{d}\,N_2}{\mathrm{d}\,\tau} &= r\,N_2\left(1-\dfrac{N_2+\alpha_1\,N_1}{K}\right)-\dfrac{m}{1+S\,N_2}\,N_2 \end{cases} \tag{1}$$

The two species have the same carrying capacity K, the same growth rate r, the same mortality rate m and the same density-dependence factor S.

Following Williams and Banyikwa (1981), these equations are equivalent to:

$$\begin{cases} \dfrac{\mathrm{d}\,N_1}{\mathrm{d}\,\tau} &= \left(r-\dfrac{m}{1+S\,N_1}\right)N_1\left(1-\dfrac{N_1}{K\left(1-\frac{m}{r\,(1+S\,N_1)}\right)}\right) \\ \dfrac{\mathrm{d}\,N_2}{\mathrm{d}\,\tau} &= \left(r-\dfrac{m}{1+S\,N_2}\right)N_2\left(1-\dfrac{N_2+\alpha_1\,N_1}{K\left(1-\frac{m}{r\,(1+S\,N_2)}\right)}\right) \end{cases} \tag{2}$$

which is the usual form of Lotka-Volterra model. This form of the systems make clear that the term of conspecific positive density dependence changes the effective carrying capacities, $K_{\mathrm{eff}_i}=K\left(1-\frac{m}{r\,(1+S\,N_i)}\right)$ (as discussed by Holt 1985, in the case of density-independent mortality).

2 Scaling

All variables are rescaled to growth rate and carrying capacity, as follows: $n_1=N_1/K$, rescaled density of species 1; $n_2=N_2/K$, rescaled density of species 2; $d=m/r$, rescaled mortality; $s=S\,K$, rescaled density-dependence factor; $t=\tau\,r$, rescaled time unit. The rescaled variables are dimensionless. From Equation 1 above, the dynamical system becomes:

$$\begin{cases} \dfrac{\mathrm{d}\,n_1}{\mathrm{d}\,t} &= n_1\left[1-n_1-\dfrac{d}{1+s\,n_1}\right] \\ \dfrac{\mathrm{d}\,n_2}{\mathrm{d}\,t} &= n_2\left[1-n_2-\alpha_1\,n_1-\dfrac{d}{1+s\,n_2}\right] \end{cases} \tag{3}$$

This is the system of equations analyzed (Equations 1a and 1b in the main text). Equations 2 and 3 in the main text are scaled in a similar manner.

Supplementary file 1B:
Analytical derivation of the two-species model with asymmetric competition for resources

I analyze the two-species model with positive density-dependent mortality and asymmetric competition for resources. Using basic linear algebra, I find equilibria and determine their stability. I also determine the threshold value of s (s^+) above which the coexistence equilibrium can be recovered, and I prove that the least competitive species is always less abundant than the most competitive species at coexistence equilibrium. Finally, I show that the least competive species is at higher density at coexistence equilibrium if $s \to \infty$ than if $s = 0$.
Here, I assume that $0 < d < 1$ and $0 < \alpha_1 < 1$. Note however that the system is characterized by more unstable equilibria for $d > 1$; even without competitors, species may not invade if they are too rare due to high mortality at low density.

1 Differential equations

$$\begin{cases} \dfrac{\mathrm{d}\, n_1}{\mathrm{d}\, t} &= n_1 \left[1 - n_1 - \dfrac{d}{1 + s\, n_1}\right] \\ \dfrac{\mathrm{d}\, n_2}{\mathrm{d}\, t} &= n_2 \left[1 - n_2 - \alpha_1\, n_1 - \dfrac{d}{1 + s\, n_2}\right] \end{cases} \tag{4}$$

with: $0 < d < 1$, $0 < \alpha_1 < 1$ and $s > 0$.

2 Equilibria

Complete Extinction

$$n_1^* = n_2^* = 0 \tag{5}$$

Only species 1

$$\begin{cases} n_1^* &= \dfrac{(s-1) + \sqrt{(s-1)^2 + 4\, s\,(1-d)}}{2\, s} > 0 \\ n_2^* &= 0 \end{cases} \tag{6}$$

Only species 2

$$\begin{cases} n_1^* &= 0 \\ n_2^* &= \dfrac{(s-1) + \sqrt{(s-1)^2 + 4\, s\,(1-d)}}{2\, s} > 0 \end{cases} \tag{7}$$

Coexistence n°1

$$\begin{cases} n_1^* &= \dfrac{(s-1) + \sqrt{(s-1)^2 + 4\, s\,(1-d)}}{2\, s} > 0 \\ n_2^*\,[n_1^*] &= \dfrac{s\,(1 - \alpha_1\, n_1^*) - 1 + \sqrt{\Delta[n_1^*]}}{2\, s} \end{cases} \tag{8}$$

with:

$$\Delta[n_1^*] = \left(s\,(1 - \alpha_1\, n_1^*) - 1\right)^2 + 4\, s\; (1 - \alpha_1\, n_1^* - d) \geq 0 \tag{9}$$

Coexistence n°2

$$\begin{cases} n_1^* &= \dfrac{(s-1) + \sqrt{(s-1)^2 + 4\, s\,(1-d)}}{2\, s} > 0 \\ n_2^*\,[n_1^*] &= \dfrac{s\,(1 - \alpha_1\, n_1^*) - 1 - \sqrt{\Delta[n_1^*]}}{2\, s} \end{cases} \tag{10}$$

3 Preliminary calculations

$1 - \alpha_1 n_1^* - d$ at coexistence equilibrium n°1 or n°2:

If $n_1^* = \frac{(s-1)+\sqrt{(s-1)^2+4\,s\,(1-d)}}{2\,s}$ at coexistence equilibrium, then:

$$\boxed{\begin{array}{l} 1-\alpha_1\,n_1^*-d<0,\ \text{for}\ s>\dfrac{\alpha_1\,(1-\alpha_1)}{\alpha_1+d-1}\ \text{and}\ \alpha_1+d-1>0 \\ 1-\alpha_1\,n_1^*-d\geq 0,\ \text{otherwise} \end{array}} \tag{11}$$

Calculation:
Given that $1 - n_1^* - \frac{d}{1+s\,n_1^*} = 0$ (equilibrium condition), then $1 - d = n_1^*\,(s\,n_1^* + 1 - s)$ and therefore:

$$1 - \alpha_1\,n_1^* - d = n_1^*\,(s\,n_1^* + 1 - s - \alpha_1) \tag{12}$$

Given that $n_1^* > 0$, $1 - \alpha_1\,n_1^* - d$ has the same sign than $s\,n_1^* + 1 - s - \alpha_1$:

$$s\,n_1^* + 1 - s - \alpha_1 = \frac{1 - s - 2\,\alpha_1 + \sqrt{(1-s)^2 + 4\,s\,(1-d)}}{2} \tag{13}$$

From this expression, we get $s\,n_1^* + 1 - s - \alpha_1 < 0$ (and therefore $1 - \alpha_1\,n_1^* - d < 0$) if:

$$s > \frac{\alpha_1\,(1-\alpha_1)}{\alpha_1 + d - 1} \text{ and } \alpha_1 + d - 1 > 0 \tag{14}$$

$1 - 2\,n_1^* - \frac{d}{(1+s\,n_1^*)^2}$ at coexistence equilibrium n°1 or n°2:

If $n_1^* = \frac{(s-1)+\sqrt{(s-1)^2+4\,s\,(1-d)}}{2\,s}$ at coexistence equilibrium, then:

$$\boxed{1 - 2\,n_1^* - \frac{d}{(1+s\,n_1^*)^2} < 0} \tag{15}$$

Calculation:
Given that $1 - n_1^* - \frac{d}{1+s\,n_1^*} = 0$ (equilibrium condition), then $\frac{d}{(1+s\,n_1^*)^2} = \frac{(1-n_1^*)^2}{d}$ and therefore:

$$1 - 2\,n_1^* - \frac{d}{(1+s\,n_1^*)^2} = 1 - 2\,n_1^* - \frac{(1-n_1^*)^2}{d} = \frac{-{n_1^*}^2 + 2\,(1-d)\,n_1^* - (1-d)}{d} = f(n_1^*) \tag{16}$$

Function f is a concave polynomial function, which maximum is negative: $f'(1-d) = 0$ and $f(1-d) = -(1-d) < 0$. Therefore, function f is negative and: $1 - 2\,n_1^* - \frac{d}{(1+s\,n_1^*)^2} < 0$

4 Conditions of existence of the coexistence equilibrium n°1

The conditions of existence of the coexistence equilibrium n°1 are:

$$\boxed{\begin{array}{l} \text{For } s \leq 1/d : \begin{cases} s \leq \dfrac{\alpha_1\,(1-\alpha_1)}{\alpha_1+d-1} \text{ for } \alpha_1+d-1>0 \\ s \geq 0 \quad \text{for } \alpha_1+d-1 \leq 0 \end{cases} \\ \text{For } s > 1/d : \;\; \alpha_1 < \dfrac{2\left(s+1-2\sqrt{s\,d}\right)}{s-1+\sqrt{(s-1)^2+4\,s\,(1-d)}} = A(s) \end{array}} \tag{17}$$

Calculation:

At coexistence equilibrium n°1, n_2^* is solution of $1-n_2^*-\alpha_1\,n_1^*-\frac{d}{1+s\,n_2^*}=0$, i.e., $-s\,{n_2^*}^2+(s\,(1-\alpha_1\,n_1^*)-1)\,n_2^*+(1-\alpha_1\,n_1^*-d)=0$.
n_2^* exists if the discriminant of the polynomial function is positive, i.e., $\Delta[n_1^*]=(s\,(1-\alpha_1\,n_1^*)-1)^2+4\,s\,(1-\alpha_1\,n_1^*-d)\geq 0$ leading to the condition $1-\alpha_1\,n_1^*-d\geq-\frac{(s\,(1-\alpha_1\,n_1^*)-1)^2}{4\,s}$.
$n_2^*\geq 0$ if the product of the roots of the polynomial is negative or the sum of the roots is positive (n_2^* is the highest root at coexistence equilibrium n°1). Using Vieta's formulas, this is the case if $\frac{1-\alpha_1\,n_1^*-d}{-s}\leq 0$ or $\frac{s\,(1-\alpha_1\,n_1^*)-1}{s}\geq 0$, i.e., $1-\alpha_1\,n_1^*-d\geq 0$ or $1-\alpha_1\,n_1^*-d\geq\frac{1}{s}-d$.

For $\mathbf{s \leq 1/d}$

There is a coexistence equilibrium n°1 if $1-\alpha_1\,n_1^*-d\geq 0$ and therefore if:

$$\begin{cases} s \leq \dfrac{\alpha_1\,(1-\alpha_1)}{\alpha_1+d-1} \text{ for } \alpha_1+d-1>0 \\ s \geq 0 \quad \text{for } \alpha_1+d-1 \leq 0 \end{cases} \tag{18}$$

(see preliminary calculations)

For $\mathbf{s > 1/d}$

There is a coexistence equilibrium n°1 if $1-\alpha_1\,n_1^*-d\geq-\frac{(s\,(1-\alpha_1\,n_1^*)-1)^2}{4\,s}$ and $1-\alpha_1\,n_1^*-d\geq\frac{1}{s}-d$.
We can show that for $s>1/d$, $1-\alpha_1\,n_1^*-d\geq-\frac{(s\,(1-\alpha_1\,n_1^*)-1)^2}{4\,s}$ necessarily leads to $1-\alpha_1\,n_1^*-d\geq\frac{1}{s}-d$.
If $1-\alpha_1\,n_1^*-d\geq-\frac{(s\,(1-\alpha_1\,n_1^*)-1)^2}{4\,s}$, then:

$$s^2\,(1-\alpha_1\,n_1^*)^2+2\,s\,(1-\alpha_1\,n_1^*)+(1-4\,s\,d)\geq 0 \tag{19}$$

With $X=1-\alpha_1\,n_1^*$, this is equivalent to:

$$s^2\,X^2+2\,s\,X+(1-4\,s\,d)\geq 0 \tag{20}$$

This polynomial function has only one positive root $X^+=\frac{-1+2\sqrt{s\,d}}{s}$ and therefore $X\geq X^+$.
Yet, for $s>1/d$, $X^+-\frac{1}{s}=\frac{-2+2\sqrt{s\,d}}{s}>0$, i.e. $X^+>\frac{1}{s}$.
Therefore $X>1/s$ and $1-\alpha_1\,n_1^*-d>\frac{1}{s}-d$.

For $s>1/d$, $1-\alpha_1\,n_1^*-d>-\frac{(s\,(1-\alpha_1\,n_1^*)-1)^2}{4\,s}$ is fullfilled (there is a coexistence equilibrium n°1) if $X>X^+$:

$$1-\alpha_1\,n_1^*>\frac{-1+2\sqrt{s\,d}}{s} \tag{21}$$

which is equivalent to:

$$\alpha_1<A(s) \tag{22}$$

with

$$A(s) = \frac{2\left(s+1-2\sqrt{s\,d}\right)}{s-1+\sqrt{(s-1)^2+4\,s\,(1-d)}} \tag{23}$$

Given that $s\,d < s$, $\;s+1-2\sqrt{s\,d} > X^2-2X+1$ (with $X=\sqrt{s\,d}$), which is a positive polynomial function. Therefore: $A(s) > 0$.

5 Conditions of existence of the coexistence equilibrium n°2

The conditions of existence of the coexistence equilibrium n°2 are:

$$\boxed{s > 1/d \quad \text{and} \quad \alpha_1 < A(s) \quad \text{and} \quad s > \frac{\alpha_1\,(1-\alpha_1)}{\alpha_1+d-1} \quad \text{and} \quad \alpha_1+d-1>0} \tag{24}$$

Calculation:

At coexistence equilibrium n°2, n_2^* is solution of $1-n_2^*-\alpha_1\,n_1^*-\frac{d}{1+s\,n_2^*}=0$, i.e., $-s\,{n_2^*}^2+(s\,(1-\alpha_1\,n_1^*)-1)\;n_2^*+(1-\alpha_1\,n_1^*-d)=0$.
Just like for coexistence equilibrium n°1, n_2^* exists if the discriminant of the polynomial function is positive, i.e., $\Delta[n_1^*] = (s\,(1-\alpha_1\,n_1^*)-1)^2+4\,s\;(1-\alpha_1\,n_1^*-d)\geq 0$ leading to the condition $1-\alpha_1\,n_1^*-d \geq -\frac{(s\,(1-\alpha_1\,n_1^*)-1)^2}{4\,s}$.
However, $n_2^* \geq 0$ if the product of the roots of the polynomial is positive and the sum of the roots is positive (n_2^* is the lowest root at coexistence equilibrium n°2). Using Vieta's formulas, this is the case if $\frac{1-\alpha_1\,n_1^*-d}{-s}\geq 0$ and $\frac{s\,(1-\alpha_1\,n_1^*)-1}{s}\geq 0$, i.e., $1-\alpha_1\,n_1^*-d\leq 0$ and $1-\alpha_1\,n_1^*-d \geq \frac{1}{s}-d$.

For $\mathbf{s \leq 1/d}$

$\frac{1}{s}-d \geq 0$ and therefore both $1-\alpha_1\,n_1^*-d\leq 0$ and $1-\alpha_1\,n_1^*-d\geq\frac{1}{s}-d$ cannot be fulfilled. There is no coexistence equilibrium n°2.

For $\mathbf{s > 1/d}$

There is a coexistence equilibrium n°2 if $1-\alpha_1\,n_1^*-d \geq -\frac{(s\,(1-\alpha_1\,n_1^*)-1)^2}{4\,s}$ and $1-\alpha_1\,n_1^*-d\geq\frac{1}{s}-d$, and $1-\alpha_1\,n_1^*-d\leq 0$.
We showed in the last section that for $s>1/d$, $\;1-\alpha_1\,n_1^*-d \geq -\frac{(s\,(1-\alpha_1\,n_1^*)-1)^2}{4\,s}$ necessarily leads to $1-\alpha_1\,n_1^*-d\geq\frac{1}{s}-d$.
Therefore, there is a coexistence equilibrium n°2 if $1-\alpha_1\,n_1^*-d \geq -\frac{(s\,(1-\alpha_1\,n_1^*)-1)^2}{4\,s}$ and $1-\alpha_1\,n_1^*-d\leq 0$.

For $s>1/d$, $\;1-\alpha_1\,n_1^*-d > -\frac{(s\,(1-\alpha_1\,n_1^*)-1)^2}{4\,s}$ is fullfilled (there is a coexistence equilibrium n°2) if $X > X^+$ (see previous section):

$$\alpha_1 < A(s) \tag{25}$$

with

$$A(s) = \frac{2\left(s+1-2\sqrt{s\,d}\right)}{s-1+\sqrt{(s-1)^2+4\,s\,(1-d)}} > 0 \tag{26}$$

Additionally, $1-\alpha_1\,n_1^*-d<0$ if $s>\frac{\alpha_1\,(1-\alpha_1)}{\alpha_1+d-1}$ and $\alpha_1+d-1>0$ (see preliminary calculations).

6 Local stability analysis

Jacobian matrix

$$J(n_1^*, n_2^*) = \begin{pmatrix} 1 - 2\,n_1^* - \frac{d}{(1+s\,n_1^*)^2} & 0 \\ -\alpha_1\,n_2^* & 1 - 2\,n_2^* - \alpha_1\,n_1^* - \frac{d}{(1+s\,n_2^*)^2} \end{pmatrix} \tag{27}$$

Complete Extinction: unstable equilibrium

$$J(0,0) = \begin{pmatrix} 1 - d > 0 & 0 \\ 0 & 1 - d > 0 \end{pmatrix} \tag{28}$$

Only species 1: stable equilibrium if $\mathbf{s > \frac{\alpha_1\,(1-\alpha_1)}{\alpha_1+d-1}}$ and $\mathbf{\alpha_1 + d - 1 > 0}$

$$J(n_1^*, 0) = \begin{pmatrix} 1 - 2\,n_1^* - \frac{d}{(1+s\,n_1^*)^2} & 0 \\ 0 & 1 - \alpha_1\,n_1^* - d \end{pmatrix} \tag{29}$$

First eigenvalue: $\lambda_1 = 1 - 2\,n_1^* - \frac{d}{(1+s\,n_1^*)^2} < 0$ (see preliminary calculations)

Second eigenvalue: $\lambda_2 = 1 - \alpha_1\,n_1^* - d < 0$ if $s > \frac{\alpha_1\,(1-\alpha_1)}{\alpha_1+d-1}$ and $\alpha_1 + d - 1 > 0$ (see preliminary calculations)

Therefore, for $s \leq 1/d$, the equilibrium 'only species 1' is unstable if the coexistence equilibrium exists.

For $s > 1/d$, the equilibrium 'only species 1' may be stable even if the coexistence equilibrium exists; this occur when the coexistence equilibrium n°2 exists.

Only species 2: unstable equilibrium

$$J(0, n_2^*) = \begin{pmatrix} 1 - d & 0 \\ -\alpha_1\,n_2^* & 1 - 2\,n_2^* - \frac{d}{(1+s\,n_2^*)^2} \end{pmatrix} \tag{30}$$

First eigenvalue: $\lambda_1 = 1 - d > 0$

Second eigenvalue: $\lambda_2 = 1 - 2\,n_2^* - \frac{d}{(1+s\,n_2^*)^2} < 0$ (see preliminary calculations)

Coexistence n°1: stable equilibrium

$$J(n_1^*, n_2^*) = \begin{pmatrix} 1 - 2\,n_1^* - \frac{d}{(1+s\,n_1^*)^2} & 0 \\ -\alpha_1\,n_2^* & 1 - 2\,n_2^* - \alpha_1\,n_1^* - \frac{d}{(1+s\,n_2^*)^2} \end{pmatrix} \tag{31}$$

First eigenvalue: $\lambda_1 = 1 - 2\,n_1^* - \frac{d}{(1+s\,n_1^*)^2} < 0$ (see preliminary calculations)

Second eigenvalue: $\lambda_2 = 1 - 2\,n_2^* - \alpha_1\,n_1^* - \frac{d}{(1+s\,n_2^*)^2} < 0$, under the conditions of existence.

Calculation:

Given that $1 - n_2^* - \alpha_1\,n_1^* - \frac{d}{1+s\,n_2^*} = 0$ (equilibrium condition), then $1 - n_2^* - \alpha_1\,n_1^* = \frac{d}{1+s\,n_2^*}$ and therefore:

$$\lambda_2 = \frac{d}{1+s\,n_2^*} - n_2^* - \frac{d}{(1+s\,n_2^*)^2} = \frac{-n_2^*}{(1+s\,n_2^*)^2}\left(s^2\,{n_2^*}^2 + 2\,s\,n_2^* + 1 - s\,d\right) \tag{32}$$

Given that $n_2^* > 0$, the sign of λ_2 corresponds to the sign of $-\lambda_2'$:

$$\lambda_2' = s^2\,{n_2^*}^2 + 2\,s\,n_2^* + 1 - s\,d \tag{33}$$

Using Vieta's formula, the sum of the roots of this polynomial function, $-2/s$, is negative and roots (discriminant> 0) are never both positive.

The higher root is $N_{\lambda_2'} = \frac{-1+\sqrt{s\,d}}{s}$, and $-\lambda_2' < 0$ (stable equilibrium) if $n_2^* > N_{\lambda_2'}$.

$$n_2^* - N_{\lambda_2'} = \frac{s\,(1-\alpha_1\,n_1^*) - 1 + \sqrt{\Delta[n_1^*]}}{2\,s} - \frac{-1+\sqrt{s\,d}}{s} \tag{34}$$

$$n_2^* - N_{\lambda_2'} = \frac{1}{2\,s}\left(s\,(1-\alpha_1\,n_1^*)+1-2\sqrt{s\,d}+\sqrt{\Delta[n_1^*]}\right) \tag{35}$$

$$n_2^* - N_{\lambda_2'} = \frac{1}{2\,s}\left(\sqrt{(s\,(1-\alpha_1\,n_1^*)+1)^2}-\sqrt{4\,s\,d}+\sqrt{\Delta[n_1^*]}\right) \tag{36}$$

$n_2^* - N_{\lambda_2'} > 0$ because $\sqrt{(s\,(1-\alpha_1\,n_1^*)+1)^2}-\sqrt{4\,s\,d} > 0$. Indeed, we can show that the following polynomial function is always positive under the conditions of existence of the coexistence equilibrium n°1:

$$F\,(n_1^*) = (s\,(1-\alpha_1\,n_1^*)+1)^2 - 4\,s\,d \tag{37}$$

$$F\,(n_1^*) = s^2\,(1-\alpha_1\,n_1^*)^2 + 2\,s\,(1-\alpha_1\,n_1^*) + (1-4\,s\,d) \tag{38}$$

With the variable transformation $X = s\,(1-\alpha_1\,n_1^*)$, function F is equivalent to function $\hat{F}$:

$$\hat{F}\,(X) = X^2 + 2\,X + (1-4\,s\,d) \tag{39}$$

Yet, we showed that the coexistence equilibrium n°1 exists if $\Delta[n_1^*] = (s\,(1-\alpha_1\,n_1^*)-1)^2 + 4\,s\,(1-\alpha_1\,n_1^*-d) > 0$, that we can rewrite as:

$$\hat{\Delta}[X] = (X-1)^2 + 4\,X - 4\,s\,d > 0 \tag{40}$$

$$\hat{\Delta}[X] = X^2 + 2\,X + (1-4\,s\,d) = \hat{F}\,(X) \tag{41}$$

Therefore, $\hat{F} > 0$, leading to $n_2^* - N_{\lambda_2'} > 0$ and to $-\lambda_2' < 0$.

Consequently, $\lambda_2 < 0$ and the coexistence equilibrium n°1 is stable if it exists.

Coexistence n°2: unstable equilibrium

$$J(n_1^*, n_2^*) = \begin{pmatrix} 1-2\,n_1^* - \frac{d}{(1+s\,n_1^*)^2} & 0 \\ -\alpha_1\,n_2^* & 1-2\,n_2^* - \alpha_1\,n_1^* - \frac{d}{(1+s\,n_2^*)^2} \end{pmatrix} \tag{42}$$

First eigenvalue: $\lambda_1 = 1-2\,n_1^* - \frac{d}{(1+s\,n_1^*)^2} < 0$ (see preliminary calculations)

Second eigenvalue: $\lambda_2 = 1-2\,n_2^* - \alpha_1\,n_1^* - \frac{d}{(1+s\,n_2^*)^2} > 0$, under the conditions of existence.

Calculation:

Given that $1 - n_2^* - \alpha_1\,n_1^* - \frac{d}{1+s\,n_2^*} = 0$ (equilibrium condition), then $1 - n_2^* - \alpha_1\,n_1^* = \frac{d}{1+s\,n_2^*}$ and therefore:

$$\lambda_2 = \frac{d}{1+s\,n_2^*} - n_2^* - \frac{d}{(1+s\,n_2^*)^2} = \frac{-n_2^*}{(1+s\,n_2^*)^2}\left(s^2\,{n_2^*}^2 + 2\,s\,n_2^* + 1 - s\,d\right) \tag{43}$$

Given that $n_2^* > 0$, the sign of λ_2 corresponds to the sign of $-\lambda_2'$:

$$\lambda_2' = s^2\,{n_2^*}^2 + 2\,s\,n_2^* + 1 - s\,d \tag{44}$$

Using Vieta's formula, the sum of the roots of this polynomial function, $-2/s$, is negative and roots (discriminant>0) are never both positive.

The higher root is $N_{\lambda_2'} = \frac{-1+\sqrt{s\,d}}{s}$, and $-\lambda_2' > 0$ (unstable equilibrium) if $n_2^* < N_{\lambda_2'}$.

$$n_2^* - N_{\lambda_2'} = \frac{s\,(1-\alpha_1\,n_1^*)-1-\sqrt{\Delta[n_1^*]}}{2\,s} - \frac{-1+\sqrt{s\,d}}{s} \tag{45}$$

$$n_2^* - N_{\lambda_2'} = \frac{1}{2\,s}\left(s\,(1-\alpha_1\,n_1^*)+1-2\sqrt{s\,d}-\sqrt{\Delta[n_1^*]}\right) \tag{46}$$

$$n_2^* - N_{\lambda_2'} = \frac{1}{2\,s}\,\left(\left(s\,(1-\alpha_1\,n_1^*)+1\right) - \left(2\,\sqrt{s\,d} + \sqrt{\Delta[n_1^*]}\right)\right) \tag{47}$$

$n_2^* - N_{\lambda_2'}$ has the same sign than $(s\,(1-\alpha_1\,n_1^*)+1)^2 - \left(2\,\sqrt{s\,d} + \sqrt{\Delta[n_1^*]}\right)^2$, which is equal to:

$$-\left[4\,s\,(1-\alpha_1\,n_1^*) + 4\,\sqrt{s\,d\,\Delta[n_1^*]}\right] < 0 \tag{48}$$

Therefore, $n_2^* - N_{\lambda_2'} < 0$.
Consequently, $\lambda_2 > 0$ and the coexistence equilibrium n°2 is unstable if it exists.

7 From s = 0 to s = 1/d: loss of the coexistence equilibrium n°1 if high α_1

For $s \leq 1/d$, there is no coexistence equilibrium n°1 if:

$$s > \frac{\alpha_1\,(\alpha_1 - 1)}{1-\alpha_1-d} \text{ and } \alpha_1 + d - 1 > 0 \tag{49}$$

There is a coexistence equilibrium n°1 if $s = 0$. Nonetheless, if $s \leq 1/d$, increasing s can lead to the loss of the coexistence equilibrium n°1. This occurs for strong asymetric competition. More precisely, increasing s to a value of $1/d$ can lead to extinction of the least competitive species if:

$$\boxed{\alpha_1 > \frac{-(1-d) + \sqrt{(1-d)(1+3d)}}{2\,d} > 0} \tag{50}$$

Calculation:

For $s = 1/d$, extinction of the least competitive species occurs if two conditions are fulfilled.
The first condition on α_1 is:

$$\alpha_1 > 1 - d \text{ to fulfill the condition } \alpha_1 + d - 1 > 0 \tag{51}$$

and the second condition on α_1 must fulfill $s > \frac{\alpha_1\,(\alpha_1-1)}{1-\alpha_1-d}$ at $s = 1/d$:

$$\frac{\alpha_1\,(\alpha_1 - 1)}{1-\alpha_1-d} - \frac{1}{d} < 0 \tag{52}$$

$$\frac{d\,\alpha_1^2 + (1-d)\,\alpha_1 - (1-d)}{d(1-\alpha_1-d)} < 0 \tag{53}$$

Given that $1 - \alpha_1 - d < 0$, extinction occurs at $s = 1/d$ if:

$$d\,\alpha_1^2 + (1-d)\,\alpha_1 - (1-d) > 0 \tag{54}$$

and therefore the second condition on α_1 is:

$$\alpha_1 > \frac{-(1-d) + \sqrt{(1-d)(1+3d)}}{2\,d} \tag{55}$$

We can easily show the second condition on α_1 necessarily fulfill the first condition:

$$\frac{-(1-d) + \sqrt{(1-d)(1+3d)}}{2\,d} > 1 - d \tag{56}$$

Therefore, for $s = 1/d$, extinction of the least competitive species occurs if:

$$\alpha_1 > \frac{-(1-d) + \sqrt{(1-d)(1+3d)}}{2\,d} > 0 \tag{57}$$

8 From s = 1/d to +∞: loss and recovery of the coexistence equilibrium n°1

For $s \geq 1/d$, there is a coexistence equilibrium n°1 if:

$$\alpha_1 < \frac{2\left(s+1-2\sqrt{s\,d}\right)}{s-1+\sqrt{(s-1)^2+4\,s\,(1-d)}} = A(s) \tag{58}$$

If function α_1 is decreasing, the coexistence equilibrium n°1 may vanish with increased s (the condition necessary for coexistence gets more restricted). On the contrary, if function α_1 is increasing, the coexistence equilibrium n°1 may be recovered with increased s.

More precisely, for $s > \frac{1+\sqrt{8\,d+1}+\sqrt{2}\sqrt{-8\,d^2+\sqrt{8d+1}+4\,d+1}}{4d}$ (threshold value represented by a dashed red line in **Figure 4** in the main text), the coexistence equilibrium n°1 can be recovered with increased s.

Calculation:

The derivative of function A is:

$$A'(s) = \frac{d\,(s-1)(s+1-2\sqrt{s\,d}) - \sqrt{(s-1)^2+4\,s\,(1-d)}\,\left(2\sqrt{s\,d}-d(s+1)\right)}{\left(s-1+\sqrt{(s-1)^2+4\,s\,(1-d)}\right)^2} \tag{59}$$

In the numerator, $d\,(s-1)(s+1-2\sqrt{s\,d})$ and $\sqrt{(s-1)^2+4\,s\,(1-d)}\,\left(2\sqrt{s\,d}-d(s+1)\right)$ are positive for $s \geq 1/d$. Therefore, $A'(s)$ has the same sign than:

$$\left[d\,(s-1)(s+1-2\sqrt{s\,d})\right]^2 - \left[\sqrt{(s-1)^2+4\,s\,(1-d)}\,\left(2\sqrt{s\,d}-d(s+1)\right)\right]^2 = 4\,d\,(1-d)\left[\sqrt{s\,d}\,(s+1)^3 - s\,\left[(s+1)^2+2\,d\,(s^2+1)\right]\right] \tag{60}$$

$4\,d\,(1-d)$, $\sqrt{s\,d}\,(s+1)^3$ and $s\,\left[(s+1)^2+2\,d\,(s^2+1)\right]$ are positive. Therefore, $A'(s)$ has the same sign than:

$$\left[\sqrt{s\,d}\,(s+1)^3\right]^2 - \left[s\,\left[(s+1)^2+2\,d\,(s^2+1)\right]\right]^2 = \left(s^2+2\,(1-2\,d)\,s+1\right)\left(ds^4-s^3-2(1-d)\,s^2-s+d\right) \tag{61}$$

$s^2+2\,(1-2\,d)\,s+1 > 0$ because $0 \leq d \leq 1$. Therefore, $A'(s)$ has the same sign than the polynomial function $P(s) = ds^4-s^3-2(1-d)\,s^2-s+d$. Using the math software *Maple*, we get two real roots for $0 \leq d \leq 1$:

$$s^- = \frac{1+\sqrt{8\,d+1}-\sqrt{2}\sqrt{-8\,d^2+\sqrt{8d+1}+4\,d+1}}{4d} \tag{62}$$

$$s^+ = \frac{1+\sqrt{8\,d+1}+\sqrt{2}\sqrt{-8\,d^2+\sqrt{8d+1}+4\,d+1}}{4d} \tag{63}$$

We can show that $s^- < 1/d$ and $s^+ > 1/d$:

$$s^- - \frac{1}{d} = \frac{-\sqrt{2}\sqrt{-8\,d^2+\sqrt{8d+1}+4\,d+1}\;-\;(3-\sqrt{8\,d+1})}{4d} \tag{64}$$

Because $3-\sqrt{8\,d+1} > 0$, $s^- - \frac{1}{d} < 0$.

$$s^+ - \frac{1}{d} = \frac{\sqrt{2}\sqrt{-8\,d^2+\sqrt{8d+1}+4\,d+1}\;-\;(3-\sqrt{8\,d+1})}{4d} \tag{65}$$

Because $3-\sqrt{8\,d+1} > 0$, $s^+ - \frac{1}{d}$ has the same sign than:

$$\left[\sqrt{2}\sqrt{-8\,d^2+\sqrt{8d+1}+4\,d+1}\right]^2 - \left[(3-\sqrt{8\,d+1})\right]^2 = 8\left[\sqrt{8\,d+1}-(2\,d^2+1)\right] \tag{66}$$

Therefore, $s^+ - \frac{1}{d}$ has the same sign than:

$$\left[\sqrt{8\,d+1}\right]^2 - \left[2\,d^2+1\right]^2 = 4\,d\,(1-d)\left(d^2+d+2\right) > 0 \tag{67}$$

Therefore, for $s > 1/d$, $A'(s) = 0$ for $s = s^+$.

The sign of $A'(s)$ for $\frac{1}{d} \leq s < s^+$ is the same than the sign of $\alpha_1'(1/d)$. The denominator of $\alpha_1'(1/d)$ is positive. The numerator of $\alpha_1'(1/d)$ is:

$$\text{Numerator}\left(C'(1/d)\right) = \frac{1}{d}(1-d)\left[(1-d) - d\sqrt{\frac{2}{d}+\frac{1}{d^2}-3}\right] \tag{68}$$

Given that $\frac{2}{d}+\frac{1}{d^2}-3 > 0$ for $0 \leq d \leq 1$, $\alpha_1'(1/d)$ has the same sign than:

$$(1-d)^2 - \left[d\sqrt{\frac{2}{d}+\frac{1}{d^2}-3}\right]^2 = -4\,d\,(1-d) < 0 \tag{69}$$

Therefore, for $\frac{1}{d} < s < s^+$, $A'(s) < 0$, i.e., the coexistence equilibrium n°1 may vanish with increased s.

Similarly, the sign of $A'(s)$ for $s > s^+$ is the same than the sign of $\lim_{s\to\infty} A'(s)$:

$$\lim_{s\to\infty} A'(s) = \frac{d}{2} > 0 \tag{70}$$

We can conclude that for $s > s^+$ (represented by a dashed red line in **Figure 4** in the main text), $A'(s) > 0$, i.e., the coexistence equilibrium n°1 can be recovered with increased s.

9 At coexistence equilibrium, species 2 is less abundant than species 1

Suppose that species 1 and species 2 are at equilibrium density n_1^*. Then the growth rate of species 2 is:

$$\left.\frac{\mathrm{d}\,n_2}{\mathrm{d}\,t}\right|_{\substack{n_1=n_1^*\\ n_2=n_1^*}} = n_1^*\left[1-n_1^*-\alpha_1\,n_1^*-\frac{d}{1+s\,n_1^*}\right] \tag{71}$$

Given that $1-n_1^*-\frac{d}{1+s\,n_1^*}=0$:

$$\left.\frac{\mathrm{d}\,n_2}{\mathrm{d}\,t}\right|_{\substack{n_1=n_1^*\\ n_2=n_1^*}} = n_1^*\left[-\alpha_1\,n_1^*\right] < 0 \tag{72}$$

Therefore: $n_2^* < n_1^*$

10 At coexistence equilibrium n°1, species 2 reaches a higher density if s $\to \infty$

For $s = 0$:

$$n_2^* \;\to\; (1-\alpha_1)(1-d) \tag{73}$$

and for $s \to \infty$:

$$n_2^* \;\to\; 1-\alpha_1 \;>\; (1-\alpha_1)(1-d) \tag{74}$$

Supplementary file 1C: Numerical method for analyzing the coexistence equilibrium in two-species models

Identifying the coexistence equilibrium

I run numerical simulations in Python (Release 2.7.15, 2018) on the interval of time $[0,\ 5,000]$. For each combination of parameters, I run 121 simulations with different initial species abundances (n_1^0, n_2^0), with n_1^0 and n_2^0 having values belonging to the discrete set $\{\,0,\ 0.1,\ 0.2,\ ...\ 1.0\,\}$. At the end of each simulation, the equilibrium reached is identified as a 'coexistence equilibrium' if species abundances are higher than 10^{-3}. Two coexistence equilibrium points are considered as equivalent if the Euclidean distance between the points is lower than 10^{-3}.

For all combinations of parameters tested, only one coexistence equilibrium is identified at most.

Identifying the nature of the coexistence equilibrium

The coexistence equilibrium (n_1^*, n_2^*) identified numerically is locally stable. Otherwise, it would not have been reached at the end of the simulation.

To identify if the coexistence equilibrium equilibrium is a global attractor, I run a simulation on the interval of time $[0,\ 500]$ with initial conditions $(n_1^*, 10^{-7})$, i.e., the least competitive species is very rare. At the end of the simulation, the coexistence equilibrium is considered as a *global attractor* if $n_2 > 10^{-7}$; the least competitive species is invading even if it is rare initially. Otherwise, the coexistence equilibrium is considered as a *local attractor*; the least competitive species does not invade and the coexistence equilibrium is not reached.
If the least competitive species can invade when it is rare, so does the most competitive species. Therefore, the criterion of invasion of the least competitive species is sufficient to identify the coexistence equilibrium as a global attractor.

Validity of the numerical method

To test the validity of this numerical method, I applied it to the two-species model with asymmetric competition for resources and conspecific positive density dependence acting only on mortality. I compared the output with analytical derivations. As shown in **Figure 4–Figure supplement 2**, the numerical method predicts very well the condition of existence of the coexistence equilibrium and its nature (local or global attractor). Therefore, we can confidently use this numerical method to analyze other two-species models that we cannot analyze analytically.

Supplementary file 1D:
Simulations of the multi-species models

Sampling initial communities

For each species i, $\alpha_{i,i}$ and $\alpha'_{i,i}$ are set to one. Parameters $\alpha_{j,i}$ and $\alpha'_{j,i} < 1$ ($\forall j \neq i$) therefore represents the strength of competition for resources and interspecific reproductive interference, *relative to* intraspecific interactions. Four different scenarios are considered successively.

(1) Only asymmetric competition. – All parameters linked to interspecific reproductive interference ($\alpha'_{i,j}$ with $i \neq j$) and to differences in basal mortality rates (δ_i) are set to zero. For each pair of species (i, j), $\alpha_{i,j}$ and $\alpha_{j,i}$ are drawn randomly from a uniform distribution within the range [0, 0.1].

(2) Only asymmetric reproductive interference. – I assume there is symmetric competition for resources ($\alpha_{i,j} = \alpha$ if $i \neq j$). All parameters linked to differences in basal mortality rates (δ_i) are set to zero. For each pair of species (i, j), $\alpha'_{i,j}$ and $\alpha'_{j,i}$ are drawn randomly from a uniform distribution within the range [0, 0.1].

(3) Only differences in basal mortality. – I assume there is symmetric competition for resources ($\alpha_{i,j} = \alpha$ if $i \neq j$). All parameters linked to interspecific reproductive interference ($\alpha'_{i,j}$ with $i \neq j$) are set to zero. For each species i, δ_i is drawn randomly from a uniform distribution within the range [0, 0.5].

(4) Random communities with all forms of asymmetry. – Species asymmetries act synergistically to promote species exclusion. Therefore, for clarity purpose, the maximum value for each parameter is reduced by half. For each pair of species (i, j), $\alpha_{i,j}$, $\alpha_{j,i}$, $\alpha'_{i,j}$ and $\alpha'_{j,i}$ are drawn randomly from a uniform distribution within the range [0, 0.05]. Likewise, δ_i is drawn randomly from a uniform distribution within the range [0, 0.25].

In supplementary analyses, parameters are drawn randomly from uniform distributions within other ranges; the results are qualitatively similar to those obtained with the arbitrary ranges chosen in the main analysis (**Figure 7–Figure supplement 1**).

Numerical simulations

For each scenario and for each combination of parameters, 500 species pools of 100 species each were constructed by drawing species parameter values from appropriate probability distributions. Numerical simulations are run in Python (Release 2.7.15, 2018) on the interval of time [0, 5000]. All species were equally abundant initially, and simulations were run long enough for initial transients to dissipate. Species were declared extinct if their density fell below 10^{-3}. At the end of each simulation, the number of species remaining was recorded.

Community assembly simulations

Following Gross (2008), I repeated simulations in **Figure 7** of the main text with the modification that species were introduced one at a time instead of all at once. At every $t = 200$ time units, one of the species in the species pool was selected at random and introduced with an abundance of 10^{-2} or 10^{-5} depending on the simulation. After 100 such introductions, the simulation ran to time $t = 5,000$. Species were declared extinct if their density fell below 10^{-3}.

As shown in **Figure 7–Figure supplement 9**, this second set of simulations in which species were introduced one at a time produced different results. Species richness is lower because less competitive species (in the broad sense) may not invade the population. In particular, conspecific positive density dependence does not help produce species-rich communities when species are introduced at low density.
